# Substrate recruitment by zDHHC protein acyltransferases

**DOI:** 10.1098/rsob.210026

**Published:** 2021-04-21

**Authors:** Martin Ian P. Malgapo, Maurine E. Linder

**Affiliations:** Department of Molecular Medicine, College of Veterinary Medicine, Cornell University, Ithaca, NY, USA

**Keywords:** palmitoylation, fatty acylation, post-translational modification, zDHHC enzyme

## Abstract

Protein palmitoylation is the post-translational attachment of fatty acids, most commonly palmitate (C16 : 0), onto a cysteine residue of a protein. This reaction is catalysed by a family of integral membrane proteins, the zDHHC protein acyltransferases (PATs), so-called due to the presence of an invariant Asp–His–His–Cys (DHHC) cysteine-rich domain harbouring the catalytic centre of the enzyme. Conserved throughout eukaryotes, the zDHHC PATs are encoded by multigene families and mediate palmitoylation of thousands of protein substrates. In humans, a number of zDHHC proteins are associated with human diseases, including intellectual disability, Huntington's disease, schizophrenia and cancer. Key to understanding the physiological and pathophysiological importance of individual zDHHC proteins is the identification of their protein substrates. Here, we will describe the approaches and challenges in assigning substrates for individual zDHHCs, highlighting key mechanisms that underlie substrate recruitment.

## Graphical abstract

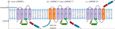

## 1. Introduction

Protein fatty acylation refers to the covalent attachment of a fatty acid to a protein. Due to the innate hydrophobic character of lipids, a common outcome of adding fatty acids to proteins is an increased binding affinity of modified proteins to biological membranes. However, protein fatty acylation can also modulate protein folding, stability and interaction with other proteins. Fatty acids of various lengths and degrees of unsaturation can be attached to a protein via a glycine (N-terminal myristoylation), lysine (*^ε^*N-fatty acylation), serine (O-fatty acylation) or cysteine (S-fatty acylation) residue [1,2]. Among these modifications, S-fatty acylation is the most prevalent [2–4]. Because the most common fatty acid donor in cells is the 16-carbon palmitoyl-Coenzyme A, the process is often referred to as palmitoylation. A key feature of palmitoylation is its reversibility. Palmitoylated substrate proteins can cycle between palmitoylated and de-palmitoylated forms on timescales that can range from seconds to hours, often in response to signals [5]. The field of protein palmitoylation has grown tremendously since the discovery of the first palmitoylated protein over 40 years ago [6]. To date, the SwissPalm database has catalogued over 10 000 unique putative palmitoylated proteins [3].

Knowledge of the mechanism by which palmitate is added to cysteine residues in proteins lagged behind the discovery of the modification, with a debate as to whether palmitate addition to protein was spontaneous or mediated by protein acyltransferases (PATs) [7]. Pioneering work from Deschenes and co-workers provided a key breakthrough. A genetic screen in yeast in the context of palmitoylation-dependent Ras signalling uncovered two genes that were required for full palmitoylation of Ras2 in *Saccharomyces cerevisiae* [8]. Named effectors of Ras function (ERF), ERF2 and ERF4 encoded proteins that formed a complex localized to the endoplasmic reticulum [9]. Purification of the Erf2/Erf4 complex enabled reconstitution of Ras2 palmitoylation *in vitro*, establishing Erf2/Erf4 as a *bona fide* PAT [10]. In parallel, a second yeast protein, ankyrin repeat (Akr) 1 protein was identified as the enzyme that mediates palmitoylation of yeast casein kinase (Yck) 2 protein [11].

A striking similarity between Erf2 and Akr1 is the presence of a highly conserved Asp–His–His–Cys (DHHC) motif within a larger cysteine-rich domain (CRD). Now confirmed as a zinc-binding domain [12–14], the zDHHC-CRD is the defining feature of an evolutionarily conserved family of zDHHC palmitoyltransferases. Genome databases annotate 7 zDHHC proteins in *S. cerevisiae* [8,11], 5 in *Schizosaccharomyces pombe* [15], 16 in *Caenorhabditis elegans* [16], 12 in *Trypanosoma brucei* [17], 23 in *Arabidopsis thaliana* [18], 22 in *Drosophila melanogaster* [19] and 23 or 24 in mammals [20,21]. Prokaryotes do not express any zDHHC enzymes, but some bacterial proteins are S-fatty acylated in a eukaryotic host [22,23].

The biomedical importance of the zDHHC enzyme family is underscored by its association with a variety of human diseases, including intellectual disability, Huntington's disease, schizophrenia and cancer [24–29]. Essential and unique roles for zDHHC proteins have been identified using zDHHC-deficient mouse models [30]. Phenotypes observed include neurodevelopmental deficits, defective learning and memory and neurodegeneration [31–34]. Accordingly, there is significant interest in understanding the mechanism and regulation of zDHHC enzymes.

This review will focus on common themes in the assignment and recruitment of palmitoylation substrates by the zDHHC PATs. We will first provide an overview of the shared structure and mechanism within this enzyme family, then outline approaches to identify substrates for individual zDHHC proteins and discuss the mechanisms that underlie substrate recruitment. For a detailed look at the structure and mechanism of zDHHC-mediated palmitoylation, the reader is referred to [35,36].

## 2. The zDHHC family of protein acyltransferases

### 2.1. Conserved sequence features

**Figure 1.**
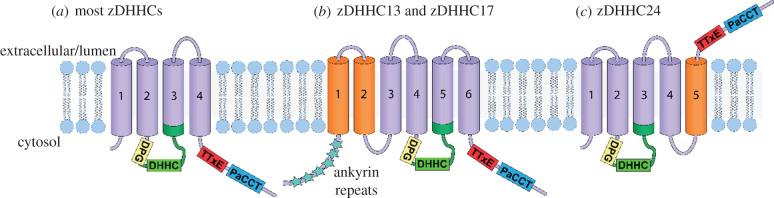
Diverse membrane topology and conserved motifs in the zDHHC enzyme family. (*a*) Most zDHHC proteins contain four TMDs. The DHHC-CRD (dark green) harbours the canonical DHHC motif (light green) situated on the cytoplasmic face of the lipid bilayer between TMD 2 and 3. Additional conserved features within the family include the DPG (yellow), TTxE (red) and PaCCT (blue) motifs. (*b*) zDHHC13 and zDHHC17 are predicted to contain six TMDs with ankyrin repeats at the N-terminus. (*c*) Sequence analysis predicts that two zDHHC proteins contain five TMDs. In zDHHC24, this implies that the C-terminus faces the lumenal side of the lipid bilayer.

All zDHHC PATs are integral membrane proteins that contain at least four transmembrane domains (TMDs) (figure 1*a*). A core topology that applies for most zDHHC proteins has the N- and C-termini and the DHHC-CRD on the cytoplasmic face of the membrane. The DHHC-CRD is located between the second and third TMDs [14]. There are variations on this organization, with three mammalian zDHHC proteins (zDHHC13, zDHHC17 and zDHHC23) predicted to contain six TMDs. Additionally, zDHHC13 and zDHHC17 contain a chain of Akrs in the N-terminus (figure 1*b*). Two zDHHC enzymes (zDHHC4 and zDHHC24) are predicted to have five TMDs. For zDHHC4, three TMDs precede the cytoplasmic DHHC-CRD, whereas in zDHHC24, the extra TMD is situated near the C-terminal domain (CTD) (figure 1*c*) [36,37].

The zDHHC enzymes are so-called due to the presence of a canonical Asp–His–His–Cys (DHHC) tetrapeptide motif that sits within a larger cysteine-rich domain (CRD) of 51-amino acid residues [38]. Together, this domain is referred to as the DHHC-CRD. Outside of the DHHC-CRD, additional conserved sequence motifs include three short conserved sequences: a Asp–Pro–Gly (DPG) motif [20], a Thr–Thr–Xxx–Glu (TTxE) motif [20] and a palmitoyltransferase conserved C-terminal (PaCCT) motif [39]. All three motifs are on the cytoplasmic face of the membrane, with the DPG motif shortly preceding the DHHC-CRD, while the TTxE and the PaCCT motifs are C-terminal to the DHHC-CRD (figure 1).

**Figure 2.**
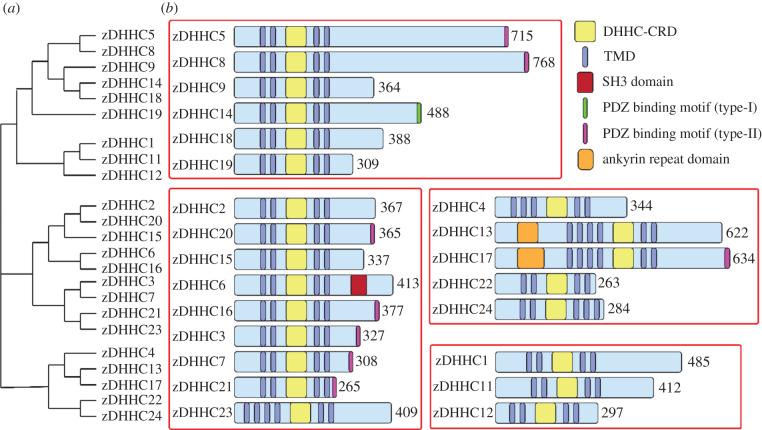
Phylogenetic analysis of human zDHHC PATs. (*a*) Phylogenetic tree of the 23 human zDHHCs PATs. The proteins were categorized into several subfamilies based on the homology of the full amino acid sequences using Clustal Omega software. (*b*) Human zDHHC PATs were grouped loosely to highlight differences in protein size and the presence of conserved binding domains and motifs.

Beyond the DHHC-CRD and conserved motifs, zDHHC PATs show significant divergence in the protein sequence. Phylogenetic analysis of the full-length sequences cluster the human zDHHC proteins into several subfamilies (figure 2). Protein size ranges from 263 amino acids for zDHHC22 to 768 for zDHHC8. Enzyme–protein substrate interaction is thought to involve residues outside of the DHHC-CRD. Known protein–protein interaction features include the Src Homology 3 (SH3) domain of zDHHC6 and the Akr domains of zDHHC13 and zDHHC17. Finally, residues within the PDZ binding motif of zDHHC5, zDHHC8 and zDHHC14 have been found to play a role in substrate recruitment [40–42]. By sequence analysis, a PDZ binding motif is predicted to function similarly in zDHHC3, zDHHC7, zDHHC16, zDHHC17, zDHHC20 and zDHHC21 [43].

### 2.2. Structure and mechanism of zDHHC PATs

**Figure 3.**
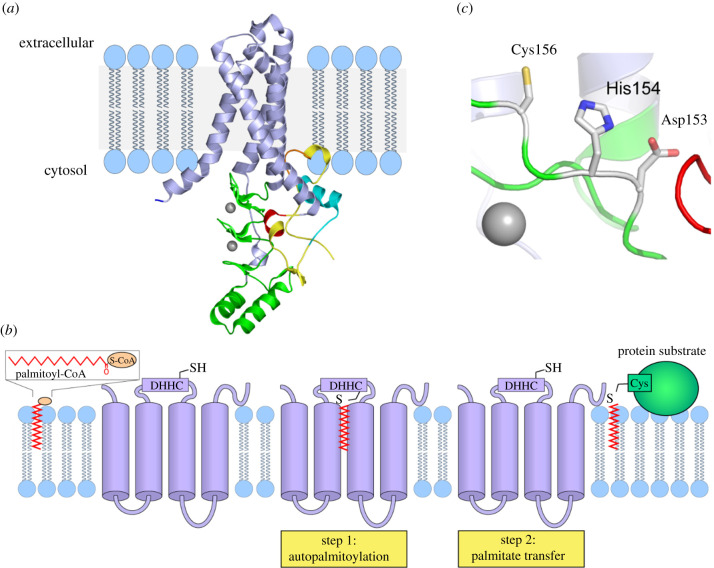
Structure and kinetic mechanism of zDHHC-mediated protein palmitoylation. (*a*) Structure of human zDHHC20 (PDB entry 6BML [14]) represented with cartoons using Pymol. TMDs 1–4 are represented as blue helices. The DHHC-CRD is represented in green, C-terminal domain (CTD) in yellow. An amphipathic helix in the CTD is represented in orange. The TTxE motif is represented in red and the PaCCT motif in cyan. The two zinc ions are represented as grey spheres. (*b*) Using palmitoyl CoA as a donor molecule, the zDHHC enzyme first modifies the cysteine of the DHHC motif with palmitate, and then transfers the palmitate from itself to the protein substrate. (*c*) The side chains involved in the catalytic triad are represented as sticks and coloured by element (C-grey, N-blue, S-yellow, O-red).

A breakthrough in the field occurred in 2018 with the report of the first x-ray crystal structures of a zDHHC enzyme. Banerjee and co-workers [14] solved the structures of human zDHHC20 and the catalytically inactive mutant of zebrafish zDHHC15. Consistent with common zDHHC protein sequence topology predictions, the four TMDs of zDHHC20 form a tepee-like cavity where the acyl chain of the acyl CoA co-substrate is inserted (figure 3*a*). The residues lining this cavity determine acyl CoA chain length selectivity. The lumenal side of the membrane contains short loops that connect TM1 and TM2, and TM3 and TM4, while the cytosolic side harbours the highly conserved DHHC-CRD connecting TM2 and TM3. The positioning of this active site at the membrane-cytosol interface is consistent with previous predictions that candidate cysteines for palmitoylation are usually proximal to the membrane [44]. Supporting previous predictions for zDHHC3 and the yeast enzyme Swf1 [12,13], the cysteine-rich domain of zDHHC20 binds two zinc ions, both adopting a tetrahedral coordination composed of three cysteines and a histidine.

The structure of human zDHHC20 demonstrates that residues in the conserved TTxE and PaCCT motifs fold within the vicinity of the DHHC-CRD and the TMDs 3–4, respectively. The second threonine and the glutamate in the TTxE motif form hydrogen bond and ionic interaction contacts with the DHHC-CRD, although the exact chemical role of these residues is currently unclear. A highly conserved Asn266 in the PaCCT motif forms extensive hydrogen bonds with neighbouring residues in TM3 and TM4 and is important for the structural integrity of the enzyme.

Following the PaCCT, there is an amphipathic helix formed by five amino acids in the CTD of zDHHC20. These residues form contacts with TM3 and TM4 and likely provide local stability. The helix is immediately followed by a hydrophobic loop that is stabilized by highly conserved lysine and proline residues. The hydrophobic residues in this loop insert into the lipid bilayer and establish additional contacts with TM2 and TM3.

The structure of zDHHC20 also sheds light on the mechanism of the zDHHC enzymes. Early kinetic analysis of yeast Erf2/Erf4 and mammalian zDHHC2 and zDHHC3 revealed that zDHHC enzymes share a two-step catalytic mechanism [45,46]. The first step, autopalmitoylation, is a fast transfer of palmitate to the zDHHC cysteine resulting in the formation of a palmitoyl–enzyme intermediate. This is followed by a slower second step, in which the palmitate moiety is transferred to the substrate protein (figure 3*b*) or hydrolysed. Mutagenesis of the DHHC cysteine suggested that the autopalmitoylated residue is the cysteine of the DHHC motif, supported by mass spectrometry of purified zDHHC3 [13] and endogenous zDHHC5, 9, 17 and 18 [47]. The crystal structure of human zDHHC20 showed alkylation on its catalytic DHHC cysteine by the common palmitoylation inhibitor 2-bromopalmitate (2-BP) [14], further demonstrating that the DHHC cysteine is the catalytic site.

In zDHHC20, the active site constitutes residues Asp153, His154 and Cys156 that form a linear catalytic triad-like arrangement (figure 3*c*). During palmitoylation, Asp153 polarizes His154 which acts a general base and deprotonates Cys156 into a thiolate. This activated cysteine then acts as nucleophile and attacks the carbonyl carbon of the fatty acyl Coenzyme A forming the DHHC-palmitate intermediate. Finally, the fatty acyl chain is transferred to the protein substrate and the DHHC enzyme is regenerated. However, alternative mechanisms for palmitoylation may be possible given previous mutagenesis studies on the DHHC motif. In Erf2, mutation of the first histidine to alanine retained enzyme autopalmitoylation but abolished palmitate transfer to Ras2 [45]. This observation suggests that the first histidine may be important for activating the protein substrate rather than the DHHC active-site cysteine. Another possible explanation is a different mechanism driven by the prerequisite of Erf2 for its binding partner Erf4 for enzymatic activity [10,35]. Interestingly, zDHHC13 uniquely possesses a natural DQHC in place of a DHHC motif. Despite having reduced autopalmitoylation relative to zDHHC17, zDHHC13 has the capacity to palmitoylate the zDHHC17 substrates huntingtin [48] and ClipR-59 [49].

Although most zDHHC proteins appear to function as PATs by themselves, there is a subset that functions with an accessory protein. Yeast Erf2 requires the formation of a heteromeric complex with its accessory protein, Erf4 for enzymatic activity toward its Ras substrate [10]. The requirement of Erf2 for Erf4 is evolutionarily conserved in *S. pombe* and mammalian orthologues [15,50,51]. The human orthologues of Erf2 and Erf4, zDHHC9 and Golga7, also referred to as GCP16, form a complex that is required for protein stability and PAT activity of zDHHC9 [51]. zDHHC5 associates with Golga7/GCP16 and the related protein Golga7b. In HEK-293T cells, complex formation between zDHHC5 and Golga7/GCP16 was shown to be independent of zDHHC5 PAT activity, but dependent on a C-terminal palmitoylation in zDHHC5 [52]. In HeLa cells, zDHHC5 palmitoylates Golga7b, and the palmitoylation and binding of Golga7b was shown to be required for the plasma membrane stabilization and substrate recruitment by zDHHC5 [53]. Finally, unrelated to the Erf4/Golga7 proteins, Selenoprotein K (SelK) has a role as an accessory protein for zDHHC6. Selk forms a complex with zDHHC6 by binding to its SH3 domain and facilitates the protein stability of the IP3 receptor, a zDHHC6 substrate [54]. For a comprehensive review of the zDHHC accessory proteins, see [55].

### 2.3. Subcellular localization

zDHHC proteins localize in multiple membrane compartments. An early study examined the intracellular localization of the ectopically expressed, epitope-tagged human and yeast zDHHC proteins [56]. The survey showed that the majority of the zDHHC proteins displayed ER and/or Golgi localization. Human zDHHC5, zDHH20 and zDHHC21, and yeast Pfa5 were found to be an exception and localized at the plasma membrane. Interestingly, yeast Pfa3 alone was found to be localized in the vacuole, and no human zDHHC protein was detected in the equivalent mammalian organelle, the lysosome. A subsequent study surveyed the localization of the murine zDHHC proteins when transfected in polarized Madin–Darby canine kidney epithelial cells [57]. An intracellular distribution of most zDHHC proteins was observed, although in this study, zDHHC5, zDHHC8 and zDHHC14 were the only zDHHC proteins localized prominently at the plasma membrane. zDHHC5 and zDHHC8 were present at lateral membranes, colocalized with their protein substrate, ankyrin-G. zDHHC14 was found on both lateral and apical membranes.

Several zDHHCs have been observed in multiple intracellular organelles. Multiple groups have found zDHHC2 in the plasma membrane and on recycling endosome [58–60]. zDHHC21 also displayed different localization patterns in various cell types: the plasma membrane in transfected HEK-293 cells [56] and the Golgi in primary keratinocytes [61]. In COS-7 cells, zDHHC21 was found predominantly in the Golgi, but still detectable in the plasma membrane [62]. Most localization studies have relied on ectopically expressed zDHHC proteins, as imaging of the endogenous proteins is limited by their low abundance and few high-quality antibodies.

Although the mechanisms that govern the spatial distribution and organelle sorting of zDHHC proteins are largely unknown, a few studies have identified sorting signals that dictate zDHHC localization. Gorleku *et al.* [63] identified and characterized lysine-based sorting signals that determine the restricted localization of zDHHC4 and zDHHC6 to ER membranes. The ER targeting signals were found to be a Lys–Xxx–Xxx (KXX) motif for zDHHC4 and a KKXX motif for zDHHC6. Adding the same targeting signal to the C-terminus of the typically Golgi-localized zDHHC3 redistributes the enzyme to the ER, signifying that the dilysine signals are sufficient to dictate ER localization. Moreover, the organelle redistribution of zDHHC3 did not affect its ability to palmitoylate its substrate synaptosome associated protein 25 (SNAP25). Another study showed that the C-terminal region of zDHHC2 and zDHHC15 regulates the localization of these two distinctly localized zDHHC PATs. zDHHC15 resides predominately in the Golgi, whereas zDHHC2 was found to cycle between endosomes and the plasma membrane. Swapping the C-terminal region of zDHHC2 to zDHHC15 altered the localization of the chimeric zDHHC15 to regions similar to that of zDHHC2 [58].

Owing to the essential roles that zDHHC PATs play in the nervous system, the subcellular localization and associated functions of these proteins in neurons has been actively investigated (for reviews, see [64–66]). Neuronal activity promotes changes in the subcellular localization of some zDHHC proteins, regulating their access to their substrates. In response to activity blockade, zDHHC2 translocates from dendritic shaft vesicles to post-synaptic densities (PSDs) at the plasma membrane of dendritic spines. This zDHHC2 translocation increases PSD95 palmitoylation in the spines leading to an upregulation of AMPA-type glutamate receptor activity, and eventually restoring homeostasis [67]. More rapid forms of plasticity are also regulated by zDHHC2 in recycling endosomes where it palmitoylates and regulates the plasticity signalling mechanisms of A-kinase anchoring protein (AKAP79/150) [68]. Increased neuronal activity disrupts the localization of endogenous zDHHC5 in synaptic membranes and enhances its internalization into dendritic shafts where it binds and palmitoylates δ-catenin. zDHHC5 is subsequently mobilized on recycling endosomes and is shuttled back into synapses with δ-catenin [69]. Taken together, these studies suggest that defined membrane targeting of active zDHHC proteins may be an important factor contributing to spatially restricted patterns of substrate palmitoylation.

There is growing appreciation that a subset of zDHHC proteins are also localized in axons. Collura *et al.* [70] examined the distribution of mammalian PATs in cultured dorsal root ganglion neurons, a widely used peripheral nervous system cell type. When transduced using lentiviral DNA, the highly related ZDHHC5 and ZDHHC8 proteins were found to be enriched in dorsal root ganglion axons where they regulate Glycoprotein 130-dependent axonal retrograde signalling. By contrast, all other PATs were detected predominantly in DRG neuronal cell bodies [70]. zDHHC14 colocalizes with its substrate PSD93 at the axon initial segment, facilitating its function to organize ion channels at this site [42].

## 3. Acyl CoA selectivity of zDHHC PATs

The most common fatty acid observed in S-fatty acylation is the saturated 16-carbon fatty acid palmitate (C16 : 0). However, S-fatty acylation of various chain lengths and degrees of unsaturation has been reported. Pioneering *in vitro* studies discovered a surprising difference in the acyl CoA specificity between zDHHC2 and zDHHC3. Whereas zDHHC2 uses a broad range of acyl CoAs, efficiently transferring acyl chains of 14 carbons or longer, zDHHC3 displays a strong preference for acyl chain lengths of 16 carbons or shorter [46]. This acyl CoA chain length selectivity in autoacylation parallels substrate specificity. A subsequent evaluation of the acyl CoA selectivity of highly related zDHHC3 and zDHHC7 in cells revealed a molecular rationale for the apparent inability of zDHHC3 to take up acyl groups bigger than 16 carbons. When expressed in mammalian cells, zDHHC3 only efficiently transfers C14 : 0, C16 : 0 and C16 : 1, while zDHHC7 prefers the longer C18 : 0. This difference is attributed to the bulkier isoleucine residue in the TM3 of zDHHC3 compared to the corresponding serine in zDHHC7 [71].

The underlying structural rationale for the acyl CoA selectivity was further nailed down in the crystal structure of zDHHC20 in which a fatty acid chain was covalently bound to its active-site cysteine. The structure showed a hydrophobic cavity formed by TM1-4 that enclosed the acyl chain. In zDHHC20, the residue that corresponds to the critical isoleucine residue in zDHHC3 is Tyr181, which forms a hydrogen bond with Ser29 at TM1. This Tyr-Ser hydrogen bond pair limits the cavity space available for the acyl CoA to insert itself. Mutation of Tyr181 to a less bulky alanine allows zDHHC20 to use the longer stearoyl-CoA (C18), while mutation of Ser29 to a bulky phenylalanine increases preference for a shorter chain acyl CoA. However, because the cavity lining residues vary significantly among different zDHHC enzymes, alternative mechanisms for acyl CoA selectivity may exist in other zDHHC enzymes [14].

It is worth noting that there is a general lack of mass spectrometry data directly confirming the molecular identity of the fatty acids attached to individual proteins. An analysis of the acyl groups in the pool of S-acylated proteins in platelets revealed that 74% were palmitate (C16 : 0), 22% were stearate (C18 : 0) and 4% were oleate (C18 : 1) [72]. Heterogeneous S-fatty acylation of Src-family kinases has been detected, with the nature of the attached fatty acid influencing raft-mediated signal transduction [73]. More recently, stearate modification of the transferrin receptor was uncovered and shown to regulate mitochondrial morphology and function [74]. The scope and functional consequences of heterogeneous S-fatty acylation of proteins, as well as how zDHHC PATs use and are regulated by acyl CoA pools is an area ripe for further investigation.

## 4. zDHHC : protein substrate assignment

### 4.1. Criteria and methods for assigning a protein substrate to a PAT

The discovery that zDHHC PATs represent multigene families posed the challenge of assigning specific substrates to individual PATs. Several criteria have been used to determine whether an authentic enzyme : substrate relationship exists. Loss or reduction of palmitoylation of a candidate substrate when expression of the cognate zDHHC protein was silenced or substantially suppressed has emerged as a principal criterion, complemented by evidence that palmitoylation of the substrate is increased when coexpressed with the PAT in cells or validated *in vitro* with purified components. Additional support for the significance of the enzyme : substrate pair can include examination of whether their spatial organization or trafficking itineraries are consistent with an interaction. Finally, confidence in the functional importance of an interaction is increased when the phenotypic consequences are the same when the palmitoylation site of a substrate is mutated, as when the zDHHC protein's activity is suppressed. Interpretation of the latter experiment may be complicated if the downregulated PAT has multiple substrates.

When attempting to assign substrates to PATs, two general approaches have been used to detect and quantify palmitoylation. The first includes variations of an acyl-exchange reaction, in which free cysteine residues are first alkylated, followed by hydroxylamine treatment to remove thioester-linked palmitate. In acyl-biotin exchange (ABE), the newly exposed cysteine thiol is reacted with a biotin-conjugated moiety that enables protein capture on avidin-based resins [75,76]. In acyl-resin assisted capture (acyl-RAC), hydroxylamine-exposed thiols are captured with thiol-reactive Sepharose resin [77]. The acyl-exchange reactions have the potential to capture all proteins with thioester linkages, but false positives arise from thioesters conjugated to other moieties, ubiquitin, for example.

The second approach uses biorthogonal click chemistry [78,79]. Cells are incubated with an alkynyl or azido fatty acid analogue, which are activated by addition to CoA, then incorporated into naturally S-acylated proteins. Via a copper-catalysed azide-alkyne cycloaddition reaction, modified proteins are conjugated to biotin, enabling pulldown by avidin-based resins. This method captures fatty acylated proteins, including proteins that are fatty acylated through linkages other than thioesters. The addition of a hydroxylamine-enrichment step to the work flow can eliminate amide- and oxyester-linked proteins from the analysis [80]. A disadvantage of this method is that it captures only proteins that are modified during the metabolic labelling period, and hence may miss proteins with slow turnover of palmitate.

### 4.2. Global approaches

Advances in methods for the detection of palmitoylated proteins in combination with mass spectrometry have resulted in the annotation of the palmitoylomes of whole organisms, cell lines and tissues. These methods have been applied to characterizing the palmitoylome in the presence or absence of a specific PAT to identify a list of candidate substrates that can be validated subsequently as bona fide substrates. The first proteomic survey of palmitoylated proteins was performed in *S. cerevisiae* using the ABE to enrich palmitoylated proteins, and quantified by spectral counts [75]. Seven genes encode zDHHC PATs in budding yeast and none are essential, enabling the construction of strains with individual deletions and combinations of deletions that could be surveyed by palmitoyl-proteomics. Over 50 proteins were identified as palmitoylated. While palmitoylation of some of these proteins was found to be dependent on specific enzymes, the knockout of individual zDHHC genes more often had only modest effect on the overall yeast palmitoylome. Interestingly, several proteins in this study maintained similar palmitoylation levels even in the absence of five of the seven yeast zDHHC proteins, suggesting a limited specificity and an extensive overlap in enzyme-substrate recognition pairs in yeast.

A number of palmitoyl-proteomic surveys have been performed to identify candidate substrates for mammalian zDHHC proteins, including zDHHC2 [81], zDHHC5 [82] and zDHHC17 [83]. These studies have revealed candidates that were subsequently validated as substrates, but there were few candidates that could be uniquely assigned to a zDHHC protein, consistent with the findings in yeast of overlapping substrate specificity. Notably, for many proteins, the reduction in palmitoylation observed for proteins paralleled a reduction in protein abundance, underscoring the importance of quantitative proteomics and consistent with evidence that for some proteins, palmitoylation is an important regulator of protein stability [84].

Advances in quantitative proteomics, particularly direct analysis of palmitoylation sites, has improved the cataloguing of palmitoylated proteins [80]. A site-specific quantitative mass spectrometry approach was applied to characterize the mouse liver palmitoylome in wild-type and zDHHC13-deficient mice. Prior studies had demonstrated that this mouse model displays severe phenotypes, including amyloidosis, alopecia and osteoporosis [33]. The finding that zDHHC13-deficient mice also have an abnormal liver function, lipid abnormalities and hypermetabolism led investigators to identify substrates that might contribute to liver pathophysiology [85]. The approach taken in this study used acyl-RAC to capture palmitoylated peptides and quantitative MS analysis that enabled site-specific identification. Application of peptide alignment between samples increased the coverage based on the retention time and *m/z* of identified peptides. To account for changes in protein expression levels that would impact quantitation of palmitoylation levels, tandem mass tagging was used to quantify protein levels for normalization. In zDHHC13-deficient mice, approximately 400 palmitoylation sites corresponding to 254 proteins were less abundant. Of these candidates, proteins associated with lipid metabolism and mitochondrial dysfunction were prominent and a number were further characterized as potential substrates. A limitation of the analysis is that the candidate proteins were validated using acyl-RAC, which as noted above, does not establish whether the thioester-linked moiety is a fatty acid.

Switching gears from a zDHHC-centric point of view to one focused on the substrate, several experimental approaches have been designed to identify the zDHHC PATs that palmitoylate a specific substrate. The first of these experiments is an overexpression strategy developed by Fukata and co-workers [86] in which each murine zDHHC PAT is coexpressed in mammalian cells with the substrate of interest. Candidate PATs are identified as those that increase labelling of the substrate with radioactive palmitate or a palmitate analogue over the level supported by endogenous zDHHC proteins [86]. These candidate enzymes are then individually knocked down to verify the legitimacy of the enzyme-substrate pair [86]. This method successfully identified an overlapping substrate specificity for zDHHC3 and zDHHC7 towards PSD95, SNAP25 and growth associated protein 43 (GAP43). Moreover, it showed that zDHHC9 and zDHHC17 had PAT activity only toward HRas and SNAP25. However, overproduction of a protein may cause it to spill out of its native compartments, as observed for the zDHHC3 enzyme. This may explain why a decrease in PSD95 palmitoylation levels was not observed in the zDHHC3 knockout mice, even though overexpression of the DHHC3 enzyme with PSD-95 was shown to increase substrate palmitoylation [87]. The mislocalized zDHHC enzyme likely retained its catalytic activity which could palmitoylate non-natural substrates resulting in false-positive enzyme-substrate pairs. Additionally, knockdown of a specific zDHHC protein does not always completely abolish substrate palmitoylation due to the palmitoylation activity being carried out by the remaining zDHHC enzymes. For example, NRas retained a low-level palmitoylation level *in vivo* even when zDHHC9 was knocked out [88].

A second screening strategy goes directly to gene silencing of individual zDHHC proteins using small-interfering RNAs [89]. An alternative to assaying substrate palmitoylation to identify the candidate PAT is to examine the consequences of silencing an individual zDHHC protein on the localization of a specific substrate. This approach was used to identify zDHHC22 and zDHHC23 as the PATs that mediate palmitoylation of the pore-forming alpha subunit of large-conductance calcium- and voltage-activated potassium (BK) channels. The first intracellular loop of this channel is palmitoylated and acts as a transplantable plasma membrane targeting sequence for a fluorescent protein, enabling the visual screen. Functionally, regulation of palmitoylation at this site within the intact BK channel is an important regulator of cell surface expression of the receptor, with zDHHC23 playing the primary role. Interestingly, the STREX variant of the BK channel includes a CTD that is palmitoylated and modified by a distinct set of zDHHC proteins [90]. Adding to this complexity is the reversibility of palmitoylation at both the first intracellular loop and the C-terminal STREX domain, where depalmitoylation is mediated by distinct acylprotein thioesterases [89,91].

Two consistent themes have emerged from studies of zDHHC proteins and their substrates. First is the broad substrate specificities of several of its member enzymes. Second is the inherent redundancies that allow for other zDHHC enzymes to palmitoylate a protein substrate in the absence of the primary zDHHC enzyme. These characteristics are likely a critical evolutionary adaptation and a hint to the complex palmitoylation events that are critical for overall cell function.

### 4.3. Protein : protein interaction motifs

Sequence elements in the N- and C-terminal domains of the zDHHC proteins contribute to substrate recruitment through protein : protein interactions. Nine human zDHHC enzymes contain short amino acid sequences at the C-terminus predicted to be ligands for PSD-95/discs large/ZO-1 (PDZ) domains, a common structural fold of 80–90 amino acids found in a variety of signalling proteins, including scaffolds that organize receptors and ion channels at synapses (figure 2) [92]. Both zDHHC5 and zDHHC8 contain a PDZ ligand in the C-terminus [93]. In zDHHC8, this motif was found to be essential for the recruitment of the substrate, protein interacting with C-kinase 1 (Pick1), whose palmitoylation is important for long-term synaptic depression in cultured mouse cerebellar Purkinje neurons [41]. Similarly, the PDZ-interacting domain of zDHHC5 is required for the palmitoylation of the neuronal PDZ domain protein, glutamate receptor-interacting protein 1 (Grip1) [40].

The mechanism of substrate recruitment by a PDZ ligand has recently been extended to zDHHC14 and the scaffold protein PSD93 [42]. zDHHC14 binds to PSD93's third PDZ domain through its C-terminal-LSSV sequence. PSD93 is a substrate for zDHHC14 as evidenced by increased PSD93 palmitoylation when the two proteins are coexpressed in heterologous cells and facilitated by an intact PDZ ligand. In hippocampal neurons, PSD93 palmitoylation is substantially reduced by gene silencing of zDHHC14. Both proteins are localized at the axon initial segment and PSD93 enrichment at this site is dependent upon its palmitoylation and the presence of zDHHC14. Taken together, these data strongly support PSD93 as a physiological substrate for PSD93. The functional importance of the interaction between zDHHC14 and PSD93 is underscored by PSD93's role as a scaffold for Kv1 potassium channel clustering and activity in hippocampal neurons. Electrophysiological analysis of outward currents and action potential firing in hippocampal neurons where zDHHC14 has been silenced strongly supports the importance of this zDHHC protein in regulating neuronal excitability [42].

Another well-studied protein fold implicated in zDHHC-substrate interactions are Akr domains, situated in the N-terminal regions of zDHHC13 and zDHHC17 (figure 2). zDHHC17, also known as huntingtin-interacting protein 14 (HIP14), binds to its substrate, huntingtin, through its Akr domains [94]. When this Akr domain was fused to the N-terminus of zDHHC3, the chimeric Akr-zDHHC3 protein was found to interact with the huntingtin protein, a property that is absent in wild-type zDHHC3. Moreover, Akr-zDHHC3 redistributed Huntingtin to the perinuclear region through palmitoylation-dependent vesicular trafficking.

The molecular details of the interaction between Akr proteins and zDHHC13/17 substrates have emerged. A previously overlooked [VIAP][VIT]XXQP motif was found in known zDHHC13 and zDHHC17 substrates including SNAP25, SNAP23, cysteine string protein and Huntingtin [95]. Crystal structures of the Akr domains of zDHHC17 and a truncated form of SNAP-25b have elucidated the nature of this interaction, attributing it primarily to hydrogen bonding and hydrophobic interactions involving the [VIAP] [VIT]XXQP motif of SNAP25b [96]. Not surprisingly, the loss of this motif in huntingtin heavily disrupted zDHHC17 binding.

Surprisingly, the literature consensus is that the catalytic DHHC-CRD plays very little role in the protein substrate specificity of DHHC enzymes. A chimeric zDHHC15 construct containing the DHHC-CRD of zDHHC3 failed to palmitoylate SNAP23, a substrate modified by zDHHC3 but not by zDHHC15, suggesting that the CRD of zDHHC3 is not sufficient to confer substrate specificity to SNAP-23 [59]. Some caution is warranted regarding this interpretation. Although the chimeric enzyme displayed a Golgi distribution similar to both DHHC3 and DHHC15, it was not clear whether the chimera retained enzyme activity, as evidenced by very weak autopalmitoylation [59]. To address whether the DHHC-CRD has a role in substrate specificity, structures of zDHHC : protein substrate complexes are an important goal for future studies, as they may reveal the potential for substrate-specific interactions with the catalytic domain.

### 4.4. PAT cascades

zDHHC proteins are palmitoylated proteins. In addition to the catalytic cysteine residue of the zDHHC motif that forms the acyl-enzyme intermediate, palmitoylation of cysteines C-terminal to the DHHC-CRD has been detected [47,97] and shown to be functionally important [47,53]. Although It was proposed that palmitoylation might be an intramolecular event, in which palmitate transfers from the DHHC cysteine to cysteines outside the CRD [97], or a homologous intermolecular transfer, there is strong evidence in at least two cases, a zDHHC PAT is palmitoylated by another [98,99].

The first such example is zDHHC16 palmitoylation of zDHHC6 [98]. Three cysteine residues within the SH3 domain of zDHHC6 are modified. Palmitoylation of zDHHC6 by itself, either in cis or in trans, was ruled out by assessing palmitoylation of a catalytically inactive zDHHC6 expressed in cells lacking the endogenous zDHHC6 enzyme. The persistence of palmitoylation in this context suggested that modification of the SH3 domain was by another enzyme. An siRNA screen and overexpression studies identified zDHHC16 as the zDHHC6 PAT. The modified cysteine residues in zDHHC6 undergo dynamic palmitoylation, with palmitate removal mediated by the acylprotein thioesterase APT2. An elegant analysis that combined site-directed mutagenesis, kinetic parameters and mathematical modelling revealed functional differences between palmitoylated species that impact protein half-life and activity. In concert with prior work from the van der Goot group on the regulation of calnexin palmitoylation [100], these studies underscore the capacity for protein palmitoylation to be finely tuned with corresponding consequences on cellular functions.

PAT activity of zDHHC6 appears to be tightly regulated by palmitoylation of the SH3 domain. Mutation of all three SH3 cysteine residues renders the enzyme inactive in cells, as assessed by palmitoylation of calnexin. By contrast, substrate palmitoylation is increased over background when zDHHC16 is overexpressed. Interestingly, unpalmitoylated zDHHC6 is the predominant form at steady state in cell lines, suggesting that too much zDHHC6 activity may be detrimental. Cellular context may be important, as higher levels of palmitoylated zDHHC6 are present in tissues [98]. The mechanism that underlies the activation of the catalytic domain by the palmitoylated SH3 domain is unknown and merits investigation.

Palmitoylation of zDHHC5 by zDHHC20 represents the second PAT cascade known to date [99]. zDHHC5 is palmitoylated at three cysteines C-terminal to the DHHC-CRD [97] and included in a predicted amphipathic α-helix [99,101]. Similar to zDHHC6, catalytically inactive zDHHC5 is palmitoylated when expressed in zDHHC5 null cells. zDHHC20 was identified as the likely upstream PAT using a proximity biotinylation approach and confirmed by a demonstration that coexpression with zDHHC5 increased its palmitoylation. Palmitoylation of the cysteine cluster in zDHHC5 is necessary for association with its substrates, Golga7b [53] and the Na-pump [99]. This facilitation of substrate recruitment would manifest as increased PAT activity. Conservation of palmitoylation sites within the PaCCT motif suggests that other PATS are subject to similar regulation.

### 4.5. Integrated mechanisms of substrate recruitment

The zDHHC structures revealed that the enzyme active site is located at the interface of the membrane and cytoplasm, appropriately positioned to modify membrane-proximal cysteines [14]. How substrate interactions with domains of the zDHHC protein that are distant from the active-site facilitate catalysis is an open question, but one that is beginning to be addressed [99]. As noted previously, the C-terminal PaCCT motif in zDHHC5 includes the palmitoylated cysteines and forms a juxtamembrane amphipathic α-helix. Palmitoylation of the zDHHC5 substrate phospholemman, a subunit of the Na-pump, was known to be dependent on this region of zDHHC5 [101]. However, the zDHHC crystal structure presented a conundrum in that the presumed PLM binding site was too distant from the active site for catalysis to occur. Through a series of complementary pulldown assays with peptides derived from zDHHC5 and PLM, Plain *et al*. demonstrated that the zDHHC5 amphipathic helix binds to the Na-pump alpha subunit. Direct binding of PLM to zDHHC5 does not occur as PLM peptides do not pulldown zDHHC5. These data suggest that substrate recruitment of PLM is indirect and mediated by the Na-pump alpha subunit. Binding of the Na-pump complex to the C-terminal region of zDHHC5 positions PLM with access to the active site in the DHHC-CRD [99].

Post-translational modifications of zDHHC5 provide an additional level of regulation of these interactions. In addition to palmitoylation at the cysteine residues within this region, a serine residue on the hydrophilic face of the helix is modified by O-GlcNAc transferase. Both palmitoylation and O-GlcNacylation of zDHHC5 support palmitoylation of PLM, and are likely to do so through increased recruitment of the Na-pump complex to zDHHC5 [99].

The C-terminal region of zDHHC5 that encompasses the palmitoylated cysteines is a ‘hot spot’ for protein interaction and post-translational modification. Notably, the Na-pump binding site overlaps with that for Golga7b [53]. Plain *et al*. [99] could not exclude the possibility of an unknown adaptor protein mediating the binding of the Na-pump to zDHHC5, but ruled out Golga7 and Golga7b as a candidate adaptor because both were absent from the proteomic survey of proteins that interact with the zDHHC5 peptide. Thus, zDHHC5 appears to function alone, as well as in conjunction with an accessory protein.

## 5. Concluding remarks

The field of protein palmitoylation has experienced rapid growth over the last 20 years due in large part to advances in chemical biology and proteomics, the discovery of the enzymes that mediate reversible palmitoylation and more recently the solution of x-ray crystal structures of zDHHC enzymes. A detailed molecular understanding of specific palmitoylation events in cells and tissues is emerging, and one informed by structure. Fine-grained mechanistic hypotheses can now be made, tested, and validated using both *in vitro* and *in vivo* systems. An immediate goal is to develop structurally precise and specific small molecule inhibitors that allow modulation of this dynamic process. Looking ahead, it will not be surprising if the next few decades see the development of drugs for therapeutic use for human diseases with a palmitoylation component.

## Data accessibility

This article does not contain any additional data.
